# Histoplasmosis Simulating Pulmonary Metastases

**DOI:** 10.1590/0037-8682-0494-2025

**Published:** 2026-02-06

**Authors:** Antônio Carlos Portugal Gomes, Gláucia Zanetti, Edson Marchiori

**Affiliations:** 1 Medimagem/BP Medicina Diagnóstica, São Paulo, SP, Brazil.; 2 Universidade Federal do Rio de Janeiro, Departamento de Radiologia, Rio de Janeiro, RJ, Brasil.

**FIGURE 1:**
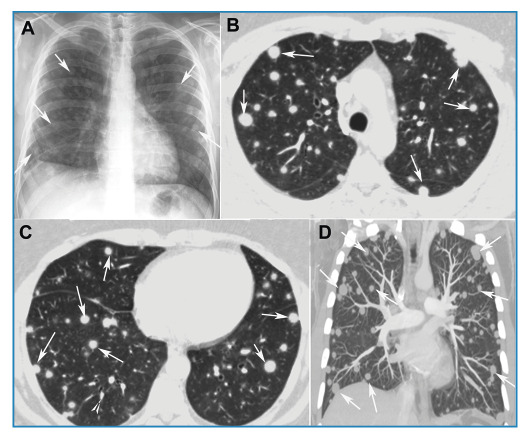
**(A)** Anteroposterior chest radiograph demonstrating multiple nodules of varying sizes in both lungs (arrows). Axial chest computed tomography images **(B)** and **(C)** and a coronal maximum-intensity projection reconstruction **(D)** showing multiple homogeneous, sharply circumscribed, randomly distributed nodules in both lungs (arrows).

**FIGURE 2:**
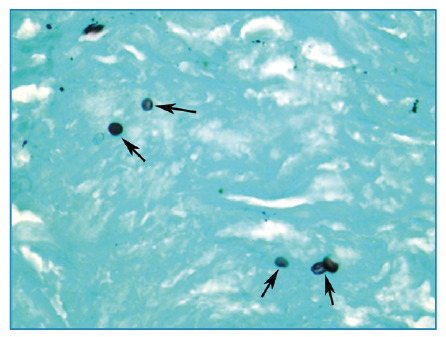
Microscopic examination of the biopsy specimen showing yeast cells of *Histoplasma capsulatum* (arrows). (Grocott, ×400).

**FIGURE 3:**
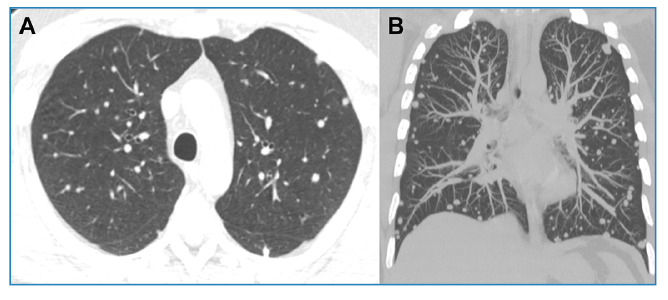
Chest computed tomography images with axial reconstruction **(A)** and coronal maximum-intensity projection reconstruction **(B)** obtained 10 months later, demonstrating a significant reduction in nodule size.

A 40-year-old immunocompetent man presented with a 30-day history of dry cough. Chest radiography revealed disseminated pulmonary nodules of various sizes (Figure 1A). Chest computed tomography (CT) showed multiple randomly distributed, homogeneous soft-tissue nodules with well-defined, regular contours (Figure 1B-D ). Laboratory test results were normal. Bronchoalveolar lavage fluid tested negative for fungi, mycobacteria, and neoplastic cells. Given these findings, pulmonary metastases were considered the main diagnostic hypothesis, and a needle biopsy of one of the pulmonary nodules was performed. Histopathological examination and culture revealed the presence of *Histoplasma capsulatum* (Figure 2). After treatment with itraconazole, follow-up CT demonstrated a significant reduction in nodule size (Figure 3). The final diagnosis of pulmonary histoplasmosis was established. The patient later reported having recently cleaned an attic containing a large amount of bat feces.

Various fungal lung infections can produce radiological findings that mimic those of malignant lung diseases (such as nodules or masses), particularly in endemic regions. Histoplasmosis is the most common fungal infection that mimics lung cancer or pulmonary metastases. In such cases, misdiagnosis leads to substantial delays in starting appropriate treatment^1^ ^-^ ^4^. When a lung infection is considered likely or possible, serological tests, sputum smears, bronchoscopy with bronchoalveolar lavage, and image-guided biopsy can be performed to aid diagnosis. Tissue specimens should be submitted not only for histopathological analysis, but also for direct examination and fungal culture^2^.

In summary, histoplasmosis should be included in the differential diagnosis of both primary and metastatic lung cancer, as early detection of pulmonary fungal disease is critical for providing proper therapy.
